# Hydroxocobalamin Dosing Strategies for Vasoplegic Syndrome Post-cardiac Surgery: A Retrospective Cohort Study

**DOI:** 10.7759/cureus.85596

**Published:** 2025-06-09

**Authors:** Quinton B Behlers, Bailey K Buenger, Gregory J Peitz, Valerie K Shostrom, Stephen C Brannan

**Affiliations:** 1 Pharmacy, Nebraska Medicine, Omaha, USA; 2 Pharmacy, Avera Heart Hospital of South Dakota, Sioux Falls, USA; 3 Biostatistics, University of Nebraska Medical Center, Omaha, USA; 4 Anesthesiology and Critical Care, University of Nebraska Medical Center, Omaha, USA

**Keywords:** cardiac surgery, cardiopulmonary bypass, catecholamine requirements, hydroxocobalamin, nitric oxide scavenging, vasoplegic syndrome

## Abstract

Introduction: Vasoplegic syndrome is a common complication following cardiac surgery, characterized by profound hypotension and high vasopressor requirements. Hydroxocobalamin, traditionally used for cyanide toxicity, has shown promise in treating refractory vasoplegic syndrome by scavenging nitric oxide. This study compares the efficacy of bolus versus extended infusion dosing strategies of hydroxocobalamin in reducing catecholamine requirements post-cardiac surgery.

Methods: We conducted a retrospective, single-center cohort study of adult patients (>18 years) admitted to the intensive care unit (ICU) after cardiac surgery between February 2020 and October 2022 who received hydroxocobalamin for vasoplegic syndrome (mean arterial pressure <65 mmHg, requiring vasopressors). Patients were grouped by hydroxocobalamin dosing: bolus (over 15 minutes) or extended infusion (over six hours). Exclusion criteria included prior methylene blue use, postoperative extracorporeal membrane oxygenation (ECMO), or intraoperative hydroxocobalamin administration. The primary outcome was the percent change in norepinephrine equivalents (NEE) over 12 hours post-administration. Secondary outcomes included NEE area under the curve (AUC), ICU and hospital length of stay, hospital survival, and additional scavenging therapy or ECMO use. Statistical analyses used Fisher’s exact test for categorical data and the Wilcoxon rank-sum test for continuous data (PC SAS v9.4, significance at p < 0.05).

Results: Of 55 patients receiving hydroxocobalamin, 13 met the inclusion criteria (eight in the bolus group and five in the extended infusion group). Baseline characteristics were similar, with 85% male patients, a median age of 61 years, and a median cardiopulmonary bypass (CPB) duration of 184 minutes (p=0.71). The percent change in NEE at 12 hours was not significantly different between groups (p=0.6084). However, the NEE AUC was significantly higher in the bolus group (median: 11.59 (interquartile range (IQR): 11.21-12.30) vs. 8.463 (IQR: 5.160-8.728), p=0.0068). Additional scavenging therapy was more frequent in the bolus group (62.5% vs. 20%, p=0.46). No differences were observed in ICU stay (23 vs. 8 days, p=0.71), hospital stay (31.5 vs. 29 days, p=0.66), or mortality (62.5% vs. 60%, p=1.0).

Conclusions: Bolus and extended infusion hydroxocobalamin dosing showed no significant difference in NEE reduction at 12 hours, though the bolus group had a higher NEE AUC, suggesting a less sustained effect. Further research is needed to optimize dosing strategies for vasoplegic syndrome post-cardiac surgery.

## Introduction

Vasoplegic syndrome is a frequent complication after cardiac surgery, with or without cardiopulmonary bypass (CPB), associated with significant morbidity and mortality (30-50%) [1,2]. It presents as a vasodilatory shock with profound systemic hypotension (mean arterial pressure (MAP) <65 mmHg), low systemic vascular resistance (<800 dyne·s/cm^5^), and elevated cardiac output (cardiac index >2.2 L/min/m²), requiring vasopressors to maintain end-organ perfusion [1].

The pathophysiology of post-CPB vasoplegic syndrome is multifactorial, involving an immunologic response with the release of pro-inflammatory mediators, complement activation, ischemia-reperfusion injury, blood transfusion, and exposure to CPB circuit surfaces [1]. This leads to elevated levels of endothelins, oxygen-free radicals, nitric oxide (NO), platelet-activating factors, thromboxane A2, cytokines, and prostaglandins [1].

Hydroxocobalamin (vitamin B12a), Food and Drug Administration (FDA)-approved for cyanide toxicity, increases arterial pressure by scavenging NO, inhibiting guanylate cyclase, and suppressing inducible NO synthase (iNOS) activity, counteracting NO-mediated vasodilation [3]. Its off-label use for refractory vasoplegic syndrome post-CPB has been reported in case series, with varying dosing strategies (bolus or extended infusion) [2,4,5]. This study compares the efficacy of bolus versus extended infusion hydroxocobalamin in reducing catecholamine requirements in vasoplegic syndrome post-cardiac surgery.

## Materials and methods

Study design

This retrospective, single-center cohort study evaluated catecholamine requirements in adult patients (>18 years) admitted to the intensive care unit (ICU) after cardiac surgery (February 2020-October 2022) who received hydroxocobalamin for vasoplegic syndrome (MAP <65 mmHg, requiring vasopressors). Patients were grouped by hydroxocobalamin administration: bolus (over 15 minutes) or extended infusion (over six hours). Exclusion criteria included prior methylene blue use, postoperative extracorporeal membrane oxygenation (ECMO) before hydroxocobalamin, or intraoperative hydroxocobalamin administration. The study was approved by the University of Nebraska Medical Center Institutional Review Board (IRB) (approval number: 0746-22-EP) with consent waived. The Office of Regulatory Affairs has confirmed that the Human Research Protection Program (HRPP) policies and institutional requirements have been met, in addition to the IRB’s approval.

Study outcomes

The primary outcome was the percent change in catecholamine requirements, measured as norepinephrine equivalents (NEE, µg/kg/min), over 12 hours post-hydroxocobalamin administration, calculated using established conversions (Table 1) [6]. Secondary outcomes included NEE area under the curve (AUC), ICU and hospital length of stay, hospital survival, and use of additional scavenging therapy or ECMO post-hydroxocobalamin.

**Table 1:** Norepinephrine equivalents NEE: norepinephrine equivalents

Drug	Dose	NEE
Epinephrine	0.1 mcg/kg/min	0.1 mcg/kg/min
Norepinephrine	0.1 mcg/kg/min	0.1 mcg/kg/min
Dopamine	15 mcg/kg/min	0.1 mcg/kg/min
Phenylephrine	1 mcg/kg/min	0.1 mcg/kg/min
Vasopressin	0.04 u/min	0.1 mcg/kg/min

Statistical analysis

Analyses were performed using PC SAS v9.4 (SAS Institute, Cary, NC, USA), with a significance level of p < 0.05. Categorical data were summarized as frequencies and percentages and compared using Fisher’s exact test. Continuous data were reported as medians and interquartile ranges (IQRs) and compared using the Wilcoxon rank-sum test.

## Results

**Figure 1:**
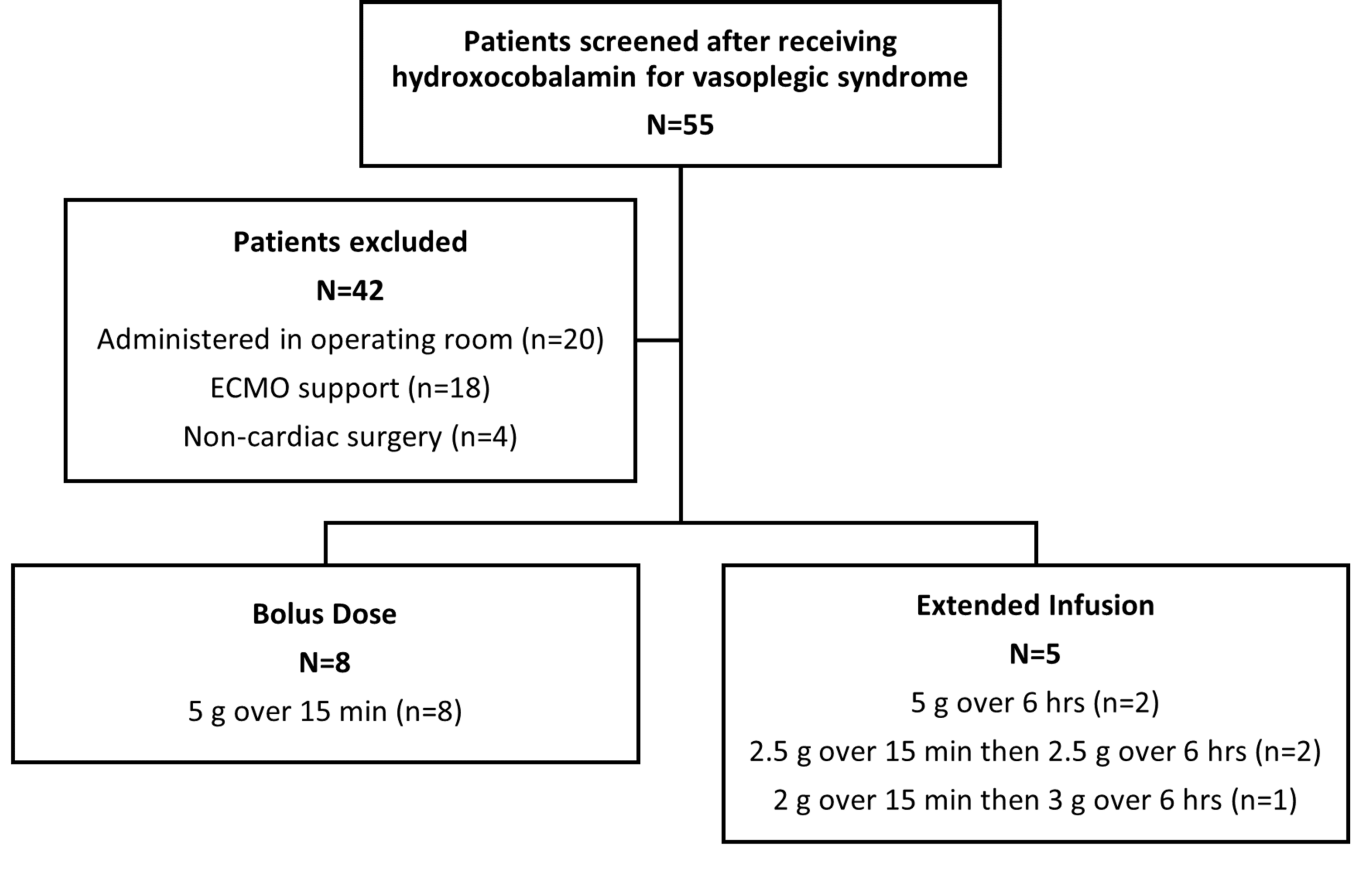
Screening flowchart ECMO: extracorporeal membrane oxygenation

Of 55 patients receiving hydroxocobalamin for vasoplegic syndrome, 13 met the inclusion criteria (Figure 1). Eight (62%) received bolus dosing, and five (38%) received extended infusion. The cohort was 85% male, with a median age of 61 years (IQR: 46.0-73.0) and a median weight of 102.3 kg (IQR: 95.5-116.6). CPB duration was similar between groups (196.5 minutes (bolus) vs. 184.0 minutes (extended infusion), p=0.71). Baseline characteristics are detailed in Table 2.

**Table 2:** Demographic and background variable ^1^Fisher Exact p-value; ^2^Wilcoxon rank sum p-value CABG: coronary artery bypass grafting; CABG valve: coronary artery bypass grafting with valve surgery; CPB: cardiopulmonary bypass; IQR: interquartile range; LVAD: left ventricular assist device

Variable	Group		Total (N=13)	p-value
	Bolus (N=8)	Extended infusion (N=5)		
Sex, n (%)				1.0000^1^
Male	7 (87.5%)	4 (80.0%)	11 (84.6%)	
Female	1 (12.5%)	1 (20.0%)	2 (15.4%)	
Weight (kg), median (IQR)	100.4 (95.0, 109.8)	104.3 (102.3, 122.6)	102.3 (95.5, 116.6)	0.5101^2^
Height (cm), median (IQR)	182.9 (177.8, 189.2)	170.2 (167.6, 182.9)	180.3 (170.2, 185.4)	
Admission age (years), median (IQR)	50.0 (35.5, 66.5)	75.0 (61.0, 79.0)	61.0 (46.0, 73.0)	0.0481^2^
CPB duration (min), median (IQR)	196.5 (145.0, 306.5)	184.0 (182.0, 192.0)	184.0 (151.0, 235.0)	0.7144^2^
Cardiac surgery, n (%)				0.3380^1^
CABG	1 (12.5%)	0 (0.0%)	1 (7.7%)	
CABG valve	1 (12.5%)	3 (60.0%)	4 (30.8%)	
Heart transplant	2 (25.0%)	2 (40.0%)	4 (30.8%)	
LVAD	3 (37.5%)	0 (0.0%)	3 (23.1%)	
Other	1 (12.5%)	0 (0.0%)	1 (7.7%)	

**Figure 2:**
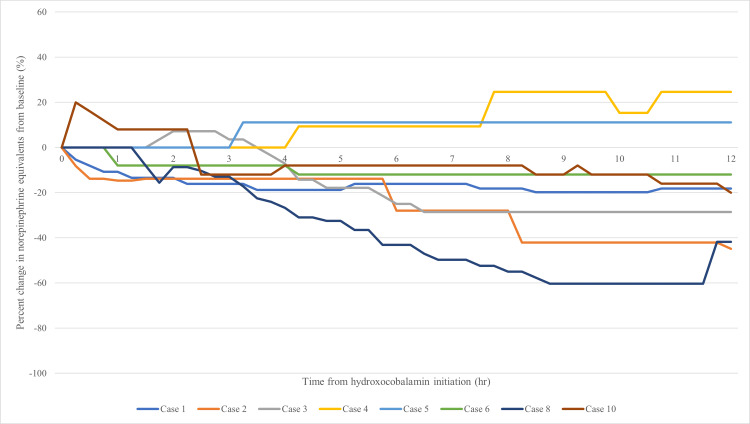
Bolus - percent change in norepinephrine equivalents from baseline

**Figure 3:**
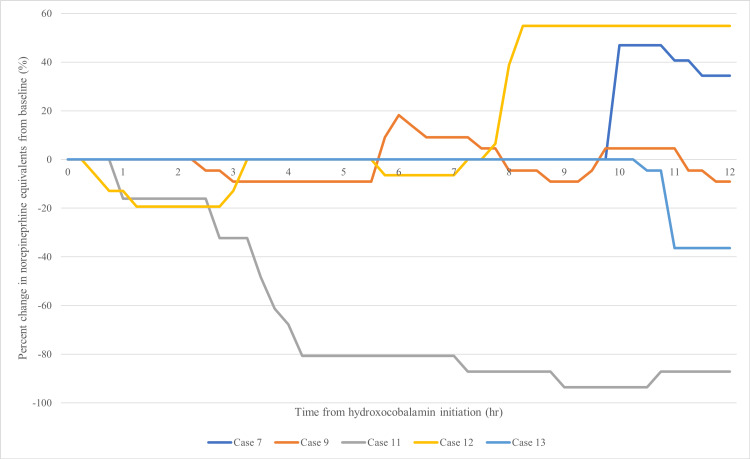
Extended infusion - percent change in norepinephrine equivalents from baseline

**Figure 4:**
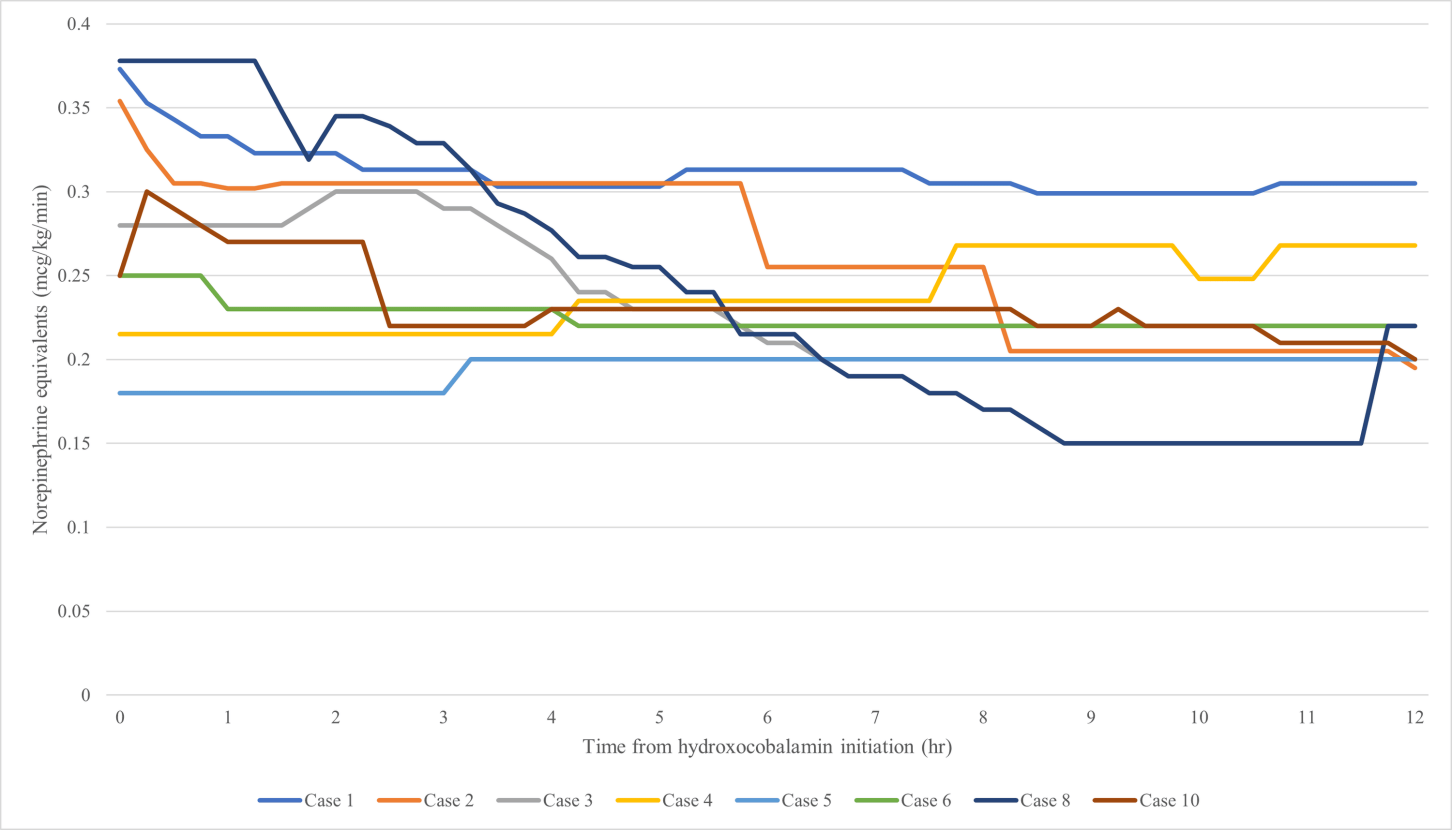
Bolus - norepinephrine equivalents

**Figure 5:**
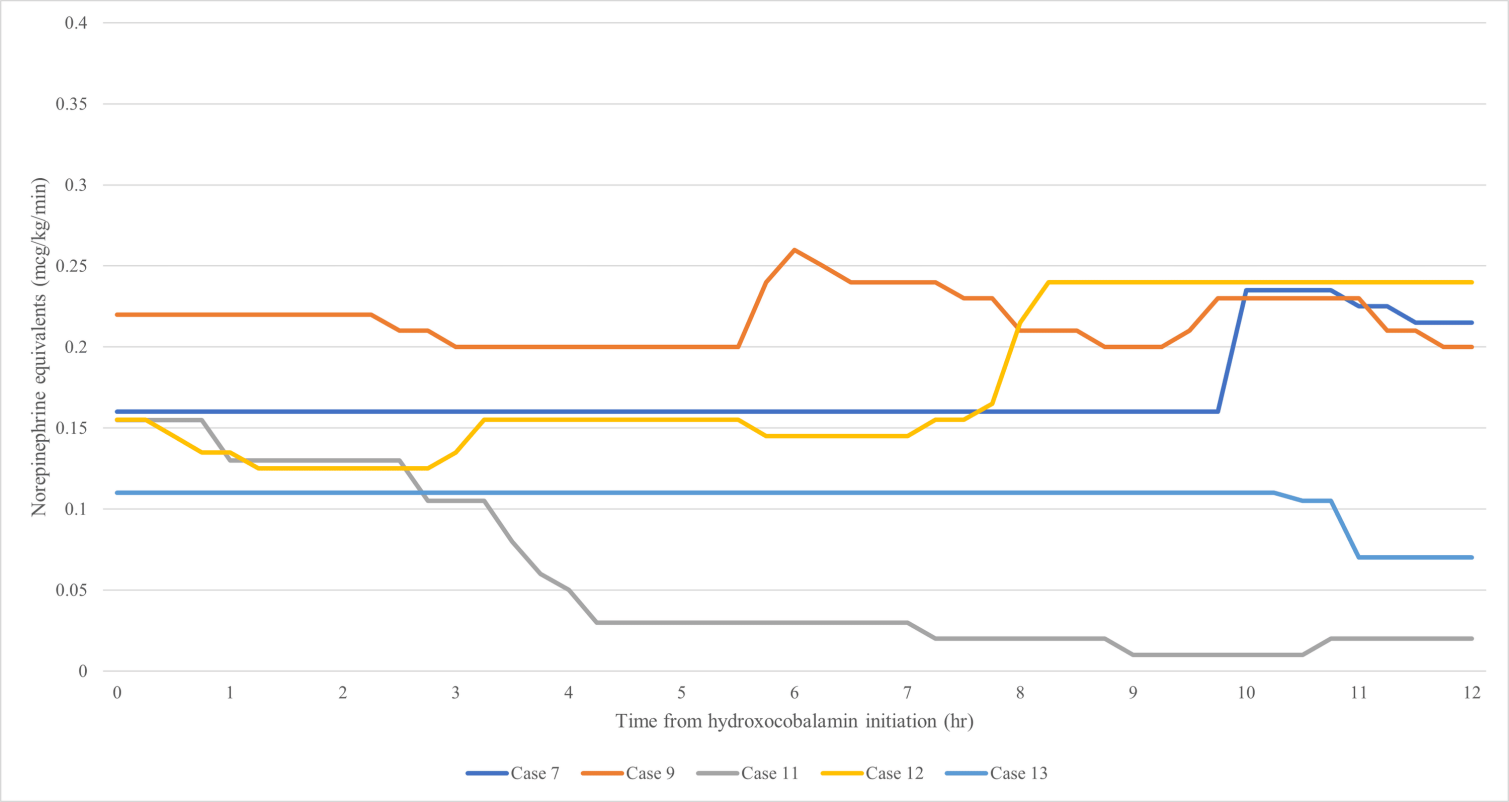
Extended infusion - norepinephrine equivalents

The percent change in NEE at 12 hours was not significantly different between groups (Table 3, p=0.6084). Graphical representations of NEE percent change and actual NEE over 12 hours are shown in Figures 2-5. The NEE AUC was significantly higher in the bolus group (median 11.59 (IQR: 11.21-12.30) vs. 8.463 (IQR: 5.160-8.728), p=0.0068, Table 4). Additional scavenging therapy was more common in the bolus group (62.5% vs. 20%, p=0.46). No significant differences were found in ICU length of stay (23 vs. 8 days, p=0.71), hospital length of stay (31.5 vs. 29 days, p=0.66), or hospital mortality (62.5% vs. 60%, p=1.0). Individual case details and secondary outcomes are provided in Tables 5-6.

**Table 3:** Percent change in norepinephrine equivalents from baseline at 12 hours

Group	N	Minimum	25th percentile	50th percentile	75th percentile	Maximum	p-value
Bolus	8	-44.9	-35.2	-19.1	-0.44	24.65	0.6084
Extended infusion	5	-87.1	-36.4	-9.09	34.38	54.84	

**Table 4:** Area under the curve of norepinephrine equivalents

Group	N	Minimum	25th percentile	50th percentile	75th percentile	Maximum	p-value
Bolus	8	9.550	11.21	11.59	12.30	15.24	0.0068
Extended infusion	5	2.657	5.160	8.463	8.728	10.62	

**Table 5:** Description of individual cases B12: vitamin B12 (hydroxocobalamin); CABG: coronary artery bypass graft; CPB: cardiopulmonary bypass; LVAD: left ventricular assist device; RVAD: right ventricular assist device; NEE: norepinephrine equivalents (µg/kg/min); Min: minimum; Max: maximum; EI: extended infusion

Case no.	Age (years)/sex	Weight (kg)	Procedure	CPB time (min)	B_12_ dosing strategy	Pre-B_12_ NEE	Post-B_12_ NEE			Hospital survival
							Min	Max	Median	
1	36/F	100.2	Heart transplant	330	5 g bolus over 15 min	0.373	0.299	0.373	0.305	Deceased
2	73/M	82.1	CABG	112	5 g bolus over 15 min	0.354	0.195	0.354	0.255	Alive
3	62/M	103	LVAD	139	5 g bolus over 15 min	0.28	0.2	0.3	0.21	Deceased
4	71/M	100.5	CABG + valve	300	5 g bolus over 15 min	0.215	0.215	0.268	0.235	Deceased
5	46/M	94.4	LVAD	151	5 g bolus over 15 min	0.18	0.18	0.2	0.2	Alive
6	31/M	95.5	LVAD	313	5 g bolus over 15 min	0.25	0.22	0.25	0.22	Deceased
7	81/M	79.1	CABG + valve	184	5 g EI over 6 hours	0.16	0.16	0.235	0.16	Deceased
8	35/M	169.7	Other: LVAD + RVAD	235	5 g bolus over 15 min	0.378	0.15	0.378	0.22	Deceased
9	61/M	122.6	Heart transplant	182	2.5 g bolus followed by 2.5 g EI over 6 hours	0.22	0.2	0.26	0.22	Deceased
10	54/M	116.6	Heart transplant	158	5 g bolus over 15 min	0.25	0.2	0.3	0.23	Alive
11	75/M	140.3	CABG + valve	204	2.5 g bolus followed by 2.5 g EI over 6 hours	0.155	0.01	0.155	0.03	Alive
12	79/F	102.3	CABG + valve	192	2 g bolus followed by 3 g EI over 6 hours	0.155	0.125	0.24	0.155	Deceased
13	58/M	104.3	Heart transplant	134	5 g EI over 6 hours	0.11	0.07	0.11	0.11	Alive

**Table 6:** Secondary outcomes ^1^Wilcoxon rank sum p-value; ^2^Fisher Exact p-value ICU: intensive care unit; IQR: interquartile range; B12: vitamin B12 (hydroxocobalamin); EI: extended infusion; NA: not applicable; ECMO: extracorporeal membrane oxygenation

Variable	Group		Total (N=13)	p-value
	Bolus (N=8)	Extended infusion (N=5)		
ICU length of stay (days), median (IQR)	23.0 (7.0, 54.0)	8.0 (7.0, 29.0)	14.0 (7.0, 53.0)	0.7144^1^
Hospital length of stay (days), median (IQR)	31.5 (17.5, 62.5)	29.0 (16.0, 41.0)	31.0 (16.0, 60.0)	0.6601^1^
Hospital mortality, n (%)				1.0000^2^
Alive	3 (37.5%)	2 (40.0%)	5 (38.5%)	
Dead	5 (62.5%)	3 (60.0%)	8 (61.5%)	
Additional scavenging therapy, n (%)				0.4615^2^
NA	3 (37.5%)	4 (80.0%)	7 (53.8%)	
B12 bolus	3 (37.5%)	0 (0.0%)	3 (23.1%)	
B12 EI	2 (25.0%)	1 (20.0%)	3 (23.1%)	
ECMO cannulation, n (%)				1.0000^2^
No	7 (87.5%)	4 (80.0%)	11 (84.6%)	
Yes	1 (12.5%)	1 (20.0%)	2 (15.4%)	

## Discussion

This retrospective cohort study is the first to directly compare bolus versus extended infusion dosing of hydroxocobalamin for vasoplegic syndrome following cardiac surgery with CPB, focusing on its impact on catecholamine requirements, quantified as NEE. The absence of a statistically significant difference in the percent change in NEE at 12 hours between the bolus and extended infusion groups suggests that both dosing strategies can reduce vasopressor needs in the short term. However, the numerically greater NEE reduction in the bolus group may reflect its use in patients with more acute and severe hemodynamic instability, where rapid NO scavenging is critical. In contrast, the extended infusion group exhibited a significantly lower NEE AUC, indicating a more sustained reduction in catecholamine requirements over 12 hours. This finding suggests that extended infusion may provide a steadier pharmacokinetic profile, potentially mitigating the rebound vasodilation seen with bolus dosing as its effects wane.

The pathophysiology of vasoplegic syndrome involves a complex interplay of inflammatory mediators and NO overproduction, driven by iNOS activation [7]. CPB triggers a systemic inflammatory response, releasing cytokines that upregulate iNOS, leading to sustained NO production independent of intracellular calcium levels [7]. Hydroxocobalamin’s mechanism - scavenging NO, inhibiting guanylate cyclase, and suppressing iNOS - directly targets this pathway [3]. Bolus dosing, by rapidly achieving high plasma concentrations, may be better suited for acute, severe vasoplegia, where NO production is rapid and overwhelming. This is supported by the clinical observation that bolus doses were often chosen for patients with acute hypotensive crises, as seen in our cohort (Table 5). Conversely, extended infusion may be more appropriate for patients with persistent, borderline vasoplegia, providing a prolonged, steady-state NO scavenging effect, as evidenced by the lower NEE AUC in this group.

Our findings align with prior studies but extend the evaluation period to 12 hours, offering new insights into the durability of hydroxocobalamin’s effects. A non-cardiac surgery study reported reduced NEE for 210 minutes in responders to bolus hydroxocobalamin [8], while a case series on extended infusion noted sustained NEE reduction for at least 600 minutes [5]. The current study’s 12-hour endpoint captures a longer timeframe, which is critical for ICU management, where sustained hemodynamic stability is paramount. The higher NEE AUC in the bolus group suggests that its rapid effect may dissipate, necessitating additional vasopressor support or scavenging therapy. This aligns with the hypothesis that bolus dosing addresses acute NO surges but may not sustain control over prolonged iNOS-driven NO production.

The vasoplegic syndrome is associated with high morbidity and mortality (30-50%) [1,2], and optimizing treatment strategies is critical. The choice of dosing strategy may depend on the clinical context: bolus dosing for rapid correction of acute hypotension and extended infusion for sustained control in less acute settings. However, the lack of an institutional protocol for hydroxocobalamin administration in our study highlights a critical gap in standardized care. Variability in dosing decisions, driven by provider preference, likely influenced outcomes and underscores the need for evidence-based guidance.

This study has several limitations. First, its retrospective, single-center design and small sample size limit the generalizability of the findings. The stringent exclusion criteria, while necessary to isolate the effects of hydroxocobalamin, reduced the sample size and excluded potentially relevant populations, such as those on ECMO or receiving intraoperative treatment. Second, the absence of a standardized protocol for vasoplegic syndrome management introduced variability in dosing and timing, potentially confounding results. Third, the study focused on postoperative ICU patients, excluding intraoperative vasoplegia, which is common during or immediately after CPB. Fourth, baseline differences in patient severity (e.g., a higher proportion of LVAD procedures in the bolus group) may have influenced outcomes, though CPB duration was similar. Finally, the high mortality rate reflects the severity of vasoplegic syndrome but complicates the interpretation of secondary outcomes like length of stay.

Future research should address these limitations through prospective, multicenter trials with larger sample sizes and standardized protocols. Investigating intraoperative hydroxocobalamin use, ECMO populations, and dose-response relationships could further refine treatment strategies.

## Conclusions

This study provides novel insights into the comparative efficacy of bolus versus extended infusion hydroxocobalamin for vasoplegic syndrome post-cardiac surgery, a condition with significant morbidity and mortality. While no significant difference was observed in the percent change in NEE at 12 hours between dosing strategies, the bolus group exhibited a higher NEE AUC, suggesting a less sustained effect compared to extended infusion. These findings highlight the potential for tailored dosing based on clinical presentation: bolus dosing for acute, severe vasoplegia and extended infusion for sustained control in persistent cases. The higher frequency of additional scavenging therapy in the bolus group further supports the hypothesis that extended infusion may offer more consistent NO scavenging over time.

Clinically, these results underscore the importance of considering both the acuity and duration of vasoplegic syndrome when selecting a dosing strategy. However, the lack of standardized protocols and the retrospective nature of this study limit definitive recommendations. The high mortality rate in both groups emphasizes the urgent need for effective therapies and highlights vasoplegic syndrome as a critical challenge in cardiac surgery. Larger, prospective studies are essential to establish optimal dosing regimens, evaluate long-term outcomes, and develop evidence-based guidance for hydroxocobalamin use in this context. Such efforts could significantly improve the management of vasoplegic syndrome, reducing morbidity and enhancing patient outcomes in the critical care setting.
